# Comparison of vilanterol, a novel long-acting beta_2_ agonist, with placebo and a salmeterol reference arm in asthma uncontrolled by inhaled corticosteroids

**DOI:** 10.1186/1477-5751-13-9

**Published:** 2014-06-13

**Authors:** Jan Lötvall, Eric D Bateman, William W Busse, Paul M O’Byrne, Ashley Woodcock, William T Toler, Loretta Jacques, Caroline Goldfrad, Eugene R Bleecker

**Affiliations:** 1 Krefting Research Centre, University of Gothenburg, Gothenburg, Sweden; 2 Department of Medicine, University of Cape Town, Cape Town, South Africa; 3 Department of Medicine, University of Wisconsin, Madison, WI, USA; 4 Michael G DeGroote School of Medicine, McMaster University, Hamilton, ON, Canada; 5 Institute of Inflammation and Repair, University of Manchester, University Hospital of South Manchester, Manchester, UK; 6 Respiratory Medicines Development Center, GlaxoSmithKline, Research Triangle Park, NC, USA; 7 Respiratory Medicines Development Centre, GlaxoSmithKline, London, UK; 8 Quantitative Sciences Division, GlaxoSmithKline, London, UK; 9 Center for Genomics and Personalized Medicine, Wake Forest University School of Medicine, Winston-Salem, NC, USA

**Keywords:** Asthma, Bronchodilators, Long-acting beta agonist, Lung function, Placebo response, Randomised trial, Salmeterol, Vilanterol

## Abstract

**Background:**

Current maintenance therapies for asthma require twice-daily dosing. Vilanterol (VI) is a novel long-acting beta_2_ agonist, under development in combination with fluticasone furoate, a new inhaled corticosteroid (ICS). Findings from a previous 4-week study suggested that VI has inherent 24-hour activity and is therefore suitable for once-daily dosing. The study described here was a double-blind, double-dummy, randomised, placebo-controlled trial, the aim of which was to assess the efficacy of once-daily VI compared with placebo in patients with persistent asthma. The primary endpoint was change from baseline in 24-hour weighted mean forced expiratory volume in 1 second after 12 weeks of treatment *vs.* placebo. An active control arm received salmeterol (SAL) twice daily. All patients were maintained on a stable background dose of ICS.

**Results:**

Patients (n = 347) received VI, placebo or SAL (1:1:1). For the primary endpoint, substantial improvements in lung function were seen with VI (359 ml), SAL (283 ml) and placebo (289 ml). There were no statistically significant treatment differences between either the VI (70 ml, *P* = 0.244) or SAL (-6 ml, *P* = 0.926) groups and placebo. Both active treatments were well tolerated, with similarly low rates of treatment-related adverse events compared with placebo. No treatment-related serious adverse events occurred.

**Conclusions:**

This study failed to show a treatment difference between VI and placebo for the primary endpoint, in the presence of a placebo response of unforeseen magnitude. Because the placebo response was so large, it is not possible to draw meaningful conclusions from the data. The reason for this magnitude of effect is unclear but it may reflect increased compliance with the anti-inflammatory therapy regimen during the treatment period.

**Trial registration:**

NCT01181895 at ClinicalTrials.gov.

## Background

Asthma is a chronic inflammatory disease characterised by airway hyper-responsiveness which causes narrowing of airways and obstruction of air flow. This typically occurs following exposure to a stimulus such as an allergen or chemical and is associated with inflammation of the airway [1]. This produces symptoms including dyspnoea (shortness of breath), wheezing and cough that generally resolve in response to treatment and/or removal of the triggering stimulus [2]. Exacerbations (‘asthma attacks’) are worsenings of symptoms associated with acute airway inflammation, and are associated with significant morbidity, mortality and healthcare costs [3].

Spirometry is a fundamental measure in the clinical management of asthma, characterising lung function and the presence of airway narrowing by assessing the degree to which airflow is limited [4]. Specifically, the forced expiratory volume in one second (FEV_1_) measurement has been validated for its close correlation with airway obstruction and is thus predictive for the presence of asthma and asthma mortality [5].

Asthma treatment is aimed at improving lung function and symptoms, along with minimising the likelihood of exacerbations [4]. Short-acting beta_2_ agonists, which provide rapid-onset relief of bronchoconstriction, are typically used on an as-needed basis and are the first line of treatment. For patients with persistent, uncontrolled asthma, maintenance therapy with inhaled corticosteroids (ICS), which treat inflammation, and long-acting beta_2_ agonists (LABAs), which improve lung function and alleviate symptoms, are recommended. Current asthma treatment guidelines [2] recommend the addition of a LABA to ICS for patients inadequately controlled by ICS monotherapy and advise against LABA monotherapy.

LABAs currently licensed for asthma, such as salmeterol (SAL) and formoterol, require twice-daily dosing. Vilanterol (VI) is chemically distinct from SAL [6] and has been shown to exhibit faster onset and longer duration of action in human lung tissue [7]. Clinical studies have demonstrated 24-hour efficacy of VI in patients with persistent asthma when administered concurrently with ICS once daily [8,9]. VI is currently under development as a once-daily treatment in combination with fluticasone furoate (FF), a novel ICS shown to be effective in a range of asthma severities [10-13].

This study sought to evaluate the efficacy and safety of once-daily VI 25 mcg over 12 weeks in patients with persistent asthma uncontrolled by ICS alone. This dose was identified from earlier-phase studies to have the greatest therapeutic ratio [8]. The main hypothesis for the study was that VI would exhibit superior efficacy relative to placebo on the primary endpoint of weighted mean (0–24 hours) FEV_1_ after 12 weeks. A SAL reference arm was also included for benchmarking.

## Results

**Figure 1:**
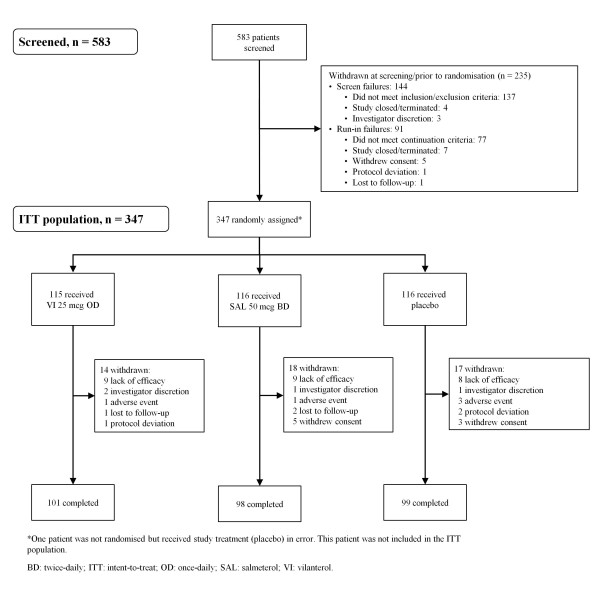
**CONSORT/patient flow diagram.** *One patient was not randomised but received study treatment (placebo) in error. This patient was not included in the ITT population. BD: twice-daily; ITT: intent-to-treat; OD: once-daily; SAL: salmeterol; VI: vilanterol.

A total of 347 patients at 34 centres in 5 countries were randomised; 298 completed the study. Patients in all treatment groups had high mean FEV_1_ reversibility (26.2–30.0%, 533.7–650.6 ml) and were symptomatic during run-in. A summary of patient disposition, including withdrawals occurring at screening, prior to randomisation and during the treatment period, is provided in Figure 1. Data describing demography (age, sex, duration of asthma, rescue use at baseline), baseline lung function (FEV_1_), reversibility and rescue medication use are provided in Table 1 and, excepting the latter, are presented by country (see Additional file 1).

**Table 1:** Patient demographics and baseline characteristics, ITT population

	**VI 25 mcg OD (N = 115)**	**SAL 50 mcg BD (N = 116)**	**Placebo (N = 116)**	**Total (N = 347)**
Age, years	41.0 (17.81)	41.1 (16.84)	41.7 (16.64)	41.3 (17.06)
Female sex, n (%)	68 (59)	77 (66)	59 (51)	204 (59)
Duration of asthma, years	17.61 (13.54)	19.41 (14.87)	18.43 (13.33)	18.49 (13.91)
Screening pre-bronchodilator FEV_1_ (ml)	2133 (631.4)	2078 (591.7)	2204 (653.0)	2139 (626.2)
Screening % predicted FEV_1_	66.6 (12.84)	65.8 (12.72)	65.9 (12.03)	66.1 (12.50)
Screening % reversibility FEV_1_	28.2 (16.36)	26.2 (13.82)	30.0 (16.58)	28.1 (15.67)
Screening absolute FEV_1_ reversibility (ml)	577.1 (344.79)	533.7 (313.85)	650.6 (383.34)	587.2 (350.84)
Baseline pre-dose FEV_1_ (ml)	2264 (619.7)	2174 (587.7)	2250 (704.0)	2229 (637.9)
Baseline rescue-free 24-hour periods (%)	7.5 (19.49)	8.0 (19.48)	4.9 (16.21)	NA
Baseline symptom-free periods (%)	5.1 (15.79)	6.0 (16.20)	2.4 (11.11)	NA

Values are mean (SD) unless otherwise stated.

BD: twice-daily; FEV_1_: forced expiratory volume in one second; ITT: intent-to-treat; OD: once-daily; SAL: salmeterol; SD: standard deviation; VI: vilanterol.

Improvements of >250 ml in the primary endpoint of weighted mean (0–24 hours) FEV_1_ were seen after 12 weeks in all treatment groups (Table 2). Neither VI nor SAL were significantly superior to placebo on the primary endpoint; a change from baseline of -6 ml compared with placebo was observed in the SAL group. As the analysis was based on a pre-defined hierarchy whereby the higher-level endpoint must be significant to infer significance for endpoints lower in the hierarchy, no statistical inference can be drawn from the observed differences for all successive endpoints.

**Table 2:** **FEV**_**1 **_**change from baseline at week 12 (day 84), ITT population**

	**VI 25 mcg OD (*n*** **= 101)**	**SAL 50 mcg BD (*n*** **= 100)**	**Placebo (*n*** **= 95)**
LS mean change from baseline (ml)	359 (41.6)	283 (41.9)	289 (42.9)
Difference *vs.* placebo (ml)	70	-6	NA
95% CI	(-48 – 188)	(-124 – 113)	
*P*-value	0.244	0.926	

Values are weighted mean (SE) 0–24 hour FEV_1_ (ml) unless otherwise stated.

ANCOVA model with covariates of baseline FEV_1_, region, age, sex and treatment.

ANCOVA: analysis of covariates; BD: twice-daily; CI: confidence interval; FEV_1_: forced expiratory volume in one second; ITT: intent-to-treat; LS: least squares; *n*: number of patients used in analysis; NA: not applicable; OD: once-daily; SAL: salmeterol; SE: standard error; VI: vilanterol.

**Figure 2:**
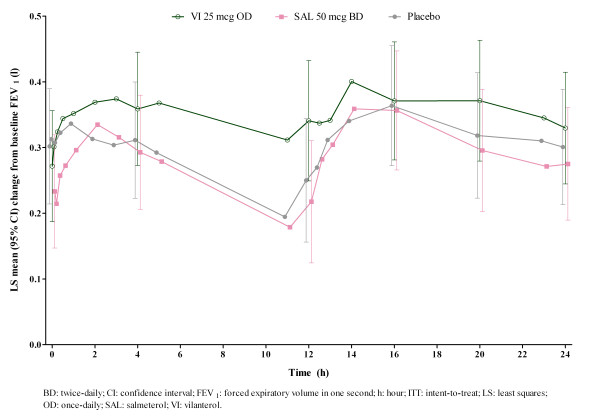
**Adjusted mean change from baseline (95% CI) in 24-hour post-dose FEV**_**1 **_**(l).** At Week 12 (Day 84), ITT population. BD: twice-daily; CI: confidence interval; FEV_1_: forced expiratory volume in one second; h: hour; ITT: intent-to-treat; LS: least squares; OD: once-daily; SAL: salmeterol; VI: vilanterol.

**Figure 3:**
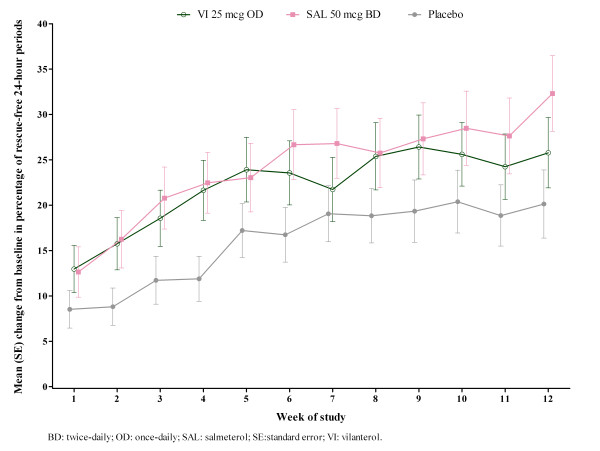
**Change from baseline in percentage of rescue-free 24-hour periods.** Over Weeks 1–12, ITT population. BD: twice-daily; OD: once-daily; SAL: salmeterol; SE: standard error; VI: vilanterol.

**Figure 4:**
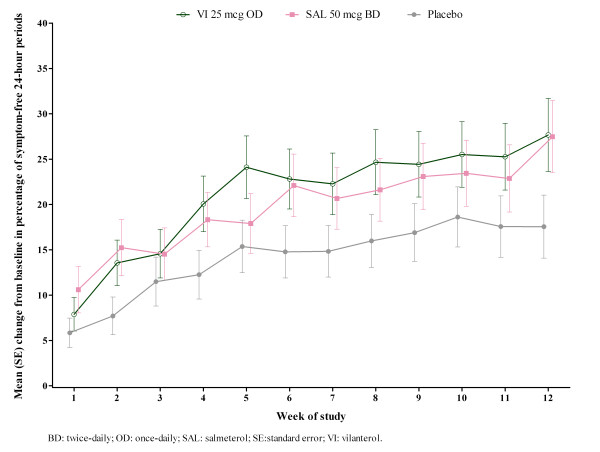
**Change from baseline in percentage of symptom-free 24-hour periods.** Over Weeks 1–12, ITT population. BD: twice-daily; OD: once-daily; SAL: salmeterol; SE: standard error; VI: vilanterol.

The relative change from baseline in raw data for weighted mean 0–24 hours serial FEV_1_ across the three treatment groups was observed to vary substantially across the five countries in which study centres were located: change from baseline after 12 weeks in the placebo group ranged from 19 ml (Germany) to 492ml (Peru) (Table 3), however, there was no evidence of a treatment interaction by region for the primary endpoint (p = 0.9178). The outcome of 0–24 hour serial FEV_1_ assessment is shown in Figure 2.Percentage of rescue-free 24-hour periods increased from baseline over the 12 weeks of treatment in all three treatment groups (VI: 21.7%, SAL: 22.9%, placebo: 14.6%) (Figure 3). Difference from placebo was marginally greater with SAL (8.3% [0.7 – 16.0]) than VI (7.1% [-0.4 – 14.6]) with some indication of a treatment-by-region interaction (p = 0.078) although the treatment differences between VI and placebo were directionally the same, favouring VI in all countries but Peru. On symptom-free 24-hour periods, change from baseline over 12 weeks relative to placebo was similar for VI (6.7% [-0.5 – 13.8]) or SAL (6.8% [-0.5 – 14.0]) (Figure 4).

**Table 3:** **FEV**_**1 **_**change from baseline at week 12 (day 84) by country, ITT population**

	**VI 25 mcg OD (*n*** **= 101)**	**SAL 50 mcg BD (*n*** **= 100)**	**Placebo (*n*** **= 95)**
Germany (N = 28)	62 (127.6)	175 (156.2)	19 (221.6)
Peru (N = 104)	391 (433.8)	360 (364.7)	492 (591.9)
Poland (N = 59)	279 (310.4)	251 (513.9)	117 (615.6)
Ukraine (N = 43)	158 (238.7)	127 (198.5)	200 (253.9)
United States (N = 62)	519 (568.2)	392 (756.0)	400 (522.5)

Values are weighted mean (SD) 0–24 hour weighted mean serial FEV_1_ (ml).

BD: twice-daily; FEV_1_: forced expiratory volume in one second; ITT: intent-to-treat; *n*: number of patients used in analysis; OD: once-daily; SAL: salmeterol; SD: standard deviation; VI: vilanterol.

Daily trough evening (PM) peak expiratory flow (PEF) increased from baseline over Weeks 1–12 in all treatment groups. For PM PEF, least squares mean change from baseline was 24.9 l/min for VI, 18.8 l/min for SAL and 11.0 l/min for placebo. The differences from placebo in mean (95% CI) change from baseline were VI: 13.9 l/min (5.1 – 22.6) and SAL: 7.8 l/min (-1.0 – 16.7). For morning (AM) PEF (l/min), increases from baseline were seen with VI: 28.0, SAL: 23.6 and placebo: 14.2. Differences from placebo were VI: 13.9 (4.8 – 22.9) and SAL: 9.5 (0.4 – 18.6) (see Additional file 2).

The median (95% CI) time to onset of a ≥12% and ≥200 ml increase from baseline FEV_1_ on Day 1 was 62 minutes in the VI group and 122 minutes in the SAL group. This could not be calculated for the placebo group because 68% of patients in the placebo group did not achieve the specified FEV_1_ increase within 2 hours and were therefore censored 2 hours post-dose. Hazard ratios for time (0–2 hours) until patients achieved an increase in FEV_1_ of ≥12% and ≥200 ml from baseline *vs.* placebo for VI and SAL on Day 1 and Day 84 are shown in Table 4.

**Table 4:** **Time to FEV**_**1 **_**increase of ≥12% and ≥200 ml from baseline, ITT population**

	**VI 25 mcg OD (N = 115)**	**SAL 50 mcg BD (N = 116)**	**Placebo (N = 116)**
**Day 1**			
*N*	115	116	113
Number (%) patients achieving threshold increase^a^	65 (57)	59 (51)	36 (32)
Hazard ratio *vs.* placebo	2.358	1.750	NA
95% CI	(1.542 – 3.606)	(1.136 – 2.696)	
**Day 84**			
*N*	101	100	96
Number (%) patients achieving threshold increase^a^	57 (56)	54 (54)	51 (53)
Hazard ratio *vs.* placebo	0.993	0.911	NA
95% CI	(0.659 – 1.495)	(0.604 – 1.374)	

^a^0–2 hours post-dose.

Cox proportional hazards model with covariates of baseline FEV_1_, sex, age, region and treatment.

BD: twice-daily; CI: confidence interval; FEV_1_: forced expiratory volume in one second; ITT: intent-to-treat; *n*: number of patients used in analysis; NA: not applicable; OD: once-daily; SAL: salmeterol; VI: vilanterol.

A statistical analysis of responders based on the Global Assessment of Change Questionnaire is presented (see Additional file 3).

Treatment with VI was well tolerated. More patients reported on-treatment adverse events (AEs) in the VI group (48%) than in the SAL (41%) or placebo (41%) groups (Table 5); the incidence of on-treatment AEs considered potentially treatment-related was low and similar across groups (VI 2%, SAL 3%, placebo 4%). Two serious AEs occurred: one fatal event in the placebo group (sudden death) and one, non-fatal event in the VI group (asthma exacerbation). Neither event was considered potentially treatment-related. No clinically significant treatment-related changes in vital signs or electrocardiogram (ECG) parameters were observed.

**Table 5:** Occurrence of all adverse events (AEs) and most frequent on-treatment AEs, ITT population

	**Number (%) of patients**		
	**VI 25 mcg OD (N = 115)**	**SAL 50 mcg BD (N = 116)**	**Placebo (N = 116)**
Any AE	55 (48)	48 (41)	47 (41)
Treatment-related AE	2 (2)	4 (3)	5 (4)
AE leading to withdrawal	2 (2)	2 (2)	3 (3)
Any serious AE	1 (<1)	0 (0)	1 (<1)
Any fatal AE	0 (0)	0 (0)	1 (<1)
**Most frequent adverse events**^**a**^			
Nasopharyngitis	9 (8)	7 (6)	12 (10)
Headache	10 (9)	9 (8)	5 (4)
Oropharyngeal pain	6 (5)	2 (2)	7 (6)
Upper respiratory tract infection	2 (2)	2 (2)	8 (7)

^a^≥5% any treatment group.

BD: twice-daily; ITT: intent-to-treat; OD: once-daily; SAL: salmeterol; VI: vilanterol.

The most frequent on-treatment AEs are listed in Table 5. Seven patients were withdrawn from the study due to AEs other than severe asthma exacerbations (VI 2, SAL 2, placebo 3). Six patients had on-treatment severe asthma exacerbations (VI 2, SAL 3, placebo 1); all received systemic/oral corticosteroids and were withdrawn from the study, with one patient in the VI arm being hospitalised. Two further patients (VI 1, SAL 1) reported severe asthma exacerbations during the post-treatment period.

There were no clinically important treatment-related changes in vital signs. A statistically, but not clinically, significant increase relative to placebo in post-dose pulse rate was seen at Week 12 with SAL (3.0 bpm; *P* = 0.013); a numerical increase was also seen with VI (2.1 bpm, *P* = 0.072). There were no apparent treatment-related changes in ECG parameters, and no clinically important liver events were recorded.

## Discussion

Current asthma treatment guidelines recommend the addition of a LABA bronchodilator to ICS anti-inflammatory therapy to provide sustained relief from narrowing of the airways [2]. LABAs currently licensed for the treatment of asthma include SAL and formoterol, and are available with fluticasone propionate and budesonide, respectively, in combination ICS/LABA formulations with duration of action approximately 12 hours [14]. As such, the currently available therapies require twice-daily dosing. VI has previously been shown to display inherent 24-hour activity [8]. In combination with the novel once-daily ICS FF, VI will potentially offer patients with persistent uncontrolled asthma a once-daily maintenance option, simplifying treatment and potentially improving patient adherence [15]. Despite the availability of effective management therapies, many patients continue to have sub-optimal control [16], in part due to failure to adhere to treatment regimens [17].

In the present study, no relative improvement in lung function compared with placebo was seen with VI or with the active comparator, SAL. Although the raw changes from baseline for VI and SAL of 359 ml and 283 ml were similar to previous findings for the same dose of VI (25 mcg) [8], the change observed with placebo (289 ml) was far higher than anticipated. The remarkable magnitude of the placebo response meant that, although substantial improvement from baseline in lung function was observed with both active treatments, it is not possible to draw meaningful conclusions about their effects on lung function or asthma symptoms from this study. Nevertheless, it is important that the findings of this study are published in order to address potential publication bias [18] and facilitate the use of the data in meta-analyses.

The wisdom of including an active reference arm to gauge assay sensitivity is clearly apparent. Previous studies performed by the sponsor have shown a clinical effect in adults and adolescents for SAL compared with placebo on lung function, however, this study did not. This supports the proposition that the reason for the failure of VI to achieve a statistically significant improvement relative to placebo is related to the unexpected and sustained improvement in the placebo group, rather than ineffectiveness of the active treatment. A possible explanation for the placebo effect, that study batches had been mis-labelled or cross-contaminated, was investigated through the re-analysis of retention samples; no evidence of incorrect treatment assignment or contamination was found. Some variation was found in the mean changes from baseline in the primary endpoint across the five countries of the study. However, despite the observed differences for improvements in lung function by country, the lack of evidence for a treatment-by-region interaction for these improvements suggests that the failure to achieve significance for the primary endpoint cannot be explained by inter-country variability.

One other possible explanation for the lack of efficacy is that there could have been an increase in patient compliance with background ICS during the study period. In order to be eligible for the study, patients were required to have been using ICS for at least 12 weeks prior to screening, with a stable ICS dose for at least 4 weeks prior to screening and during the run-in period. Patients were excluded from randomisation if they were not compliant with their ICS on at least 4 of the last 7 consecutive days of run-in. To confirm compliance, patients in this study were asked daily via the e-diary if they had used their ICS. However, real-world compliance with ICS maintenance therapy among asthma patients is often poor [19]. As such, it is conceivable that, despite the eligibility criteria requiring stable ICS use, a proportion of study participants may have only begun using their ICS regularly immediately prior to baseline. Comparatively, in a previous study of VI versus placebo in which compliance with ICS was required during the 4-week run-in period, a statistically significant improvement in lung function was seen [8].

Investigators were asked to counsel non-adherent patients on the importance of taking ICS at the prescribed dose at each clinic visit. This may have resulted in an improvement in adherence. The high degree of FEV_1_ reversibility observed among the population adds further weight to the suggestion that ICS non-adherence may have affected the outcome of this study. In future studies, confirmation of adherence during the run-in period could be addressed by providing single-blind ICS with dose counters, in order to better compare the ICS response with the observed treatment effect following the addition of vilanterol.

## Conclusions

Substantial improvements in lung function and asthma control were seen in all treatment groups, thus the unexpectedly strong placebo response confounded interpretation of the primary and secondary comparisons. As such, no firm conclusions can be drawn from these data regarding the efficacy of VI in asthma not controlled by ICS alone; however, the safety data do confirm the tolerability of VI 25 mcg.

## Materials and methods

This was a randomised, stratified, multicentre, double-blind, double-dummy (i.e. all patients received both devices, see below), parallel-group, placebo-controlled, active-controlled Phase III study of 12 weeks’ duration (GlaxoSmithKline study number: B2C112060; ClinicalTrials.gov number: NCT01181895). It was conducted between 15 September 2010 and 26 August 2011 at 34 centres in 5 countries (Germany, Peru, Poland, Ukraine and USA). The study was approved by local ethics review committees (see Additional file 4), and was conducted in accordance with the Declaration of Helsinki and Good Clinical Practice guidelines. All patients gave written informed consent.

### Eligibility criteria and interventions

In order to be eligible for screening, patients were aged ≥12 years, with a diagnosis of asthma for ≥12 weeks, and use of ICS for ≥12 weeks with stable ICS dose (200–1000 mcg fluticasone propionate or equivalent) for ≥4 weeks prior to screening. At screening, a best pre-bronchodilator FEV_1_ of 40–90% of predicted normal and demonstration of ≥12% and ≥200 ml reversibility of FEV_1_ within 10–40 minutes of rescue medication were required. To be eligible for randomisation, patients were required to be symptomatic on their current ICS treatment, defined as asthma symptom score ≥1 and/or rescue use on ≥4 of the last 7 days of the run-in, and compliant with baseline medication on ≥4 of the last 7 days of the run-in period. Compliance was assessed by a daily question of ICS compliance in the eDiary; patients were also contacted by telephone approximately two weeks after Visit 1 to assess compliance. Patients were excluded if they had a history of life-threatening asthma, or asthma exacerbation requiring systemic corticosteroids or emergency-room attendance within 3 months or overnight hospitalisation within 6 months prior to Visit 1. All patients continued the same dose of ICS throughout the treatment and follow-up periods. Patients were required to replace their current short-acting beta_2_ agonists with albuterol/salbutamol inhaler provided at Visit 1 for use as needed during the study. All systemic, oral, parenteral and depot corticosteroids were prohibited from within 12 weeks of Visit 1. All anti-leukotrienes, inhaled or oral LABA or ICS/LABA therapies, theophyllines, anticholinergics, ketotifen, nedocromil sodium and sodium cromoglycate were prohibited from Visit 1 for the duration of the study. Any other medications with the potential to affect the course of asthma or interact with sympathomimetic amines were prohibited throughout the study.

Patients were stratified according to their screening stable dose of ICS medication then randomised (1:1:1) to receive VI 25 mcg via ELLIPTA™ dry powder inhaler (representing an emitted dose from the dry powder inhaler of 22 mcg) once daily, SAL 50 mcg via Diskus®/Accuhaler® twice daily, or placebo over the duration of the study (ELLIPTA™ is a trademark of the GlaxoSmithKline group of companies). Patients and investigators were blinded to treatment assignment. All patients received double-blinded placebo dry powder inhaler and Diskus® inhalers for use as appropriate (once daily and twice daily, respectively). Inhalers containing active treatment and placebo were indistinguishable. The randomisation schedule was generated by RandAll (GlaxoSmithKline, UK) following stratification of the patients according to dose of ICS medication (low, medium or high). Patients were randomised to treatment using an automated, telephone-based Registration and Medication Ordering System (RAMOS). Treatment compliance was measured by reviewing the dose counter on the inhalers.

The intent-to-treat (ITT) population comprised all patients randomised to treatment who received at least one dose of study drug. The ITT population was used for all efficacy and safety analyses other than those specified as being carried out in the per protocol (PP) population. The PP population comprised all patients in the ITT population who did not have any full protocol deviations. Patients with only partial deviations were considered part of the PP population, but their data were excluded from the analysis from the date of the deviation onwards. The decision to exclude a patient or some of a patient’s data from the PP population was made prior to breaking the blind.

### Outcomes

The primary endpoint was change from baseline in 0–24 hours weighted mean FEV_1_ after 12 weeks. Mean change from baseline in percentage rescue-free 24-hour periods over the treatment period was a powered secondary endpoint. Other secondary endpoints were mean change from baseline in the percentage of symptom-free 24-hour periods and in individual 0–24 hours serial FEV_1_ assessments after 12 weeks. Other efficacy endpoints included change from baseline in daily trough (pre-dose, pre-rescue) PM PEF over 12 weeks of treatment, change from baseline in daily AM PEF over 12 weeks of treatment, time to increase in FEV_1_ to ≥12% and ≥200 ml above baseline on Day 1 and Day 84 (0–2 hours), and Global Assessment of Change questionnaire scores after 4 and 12 weeks of treatment. Safety endpoints included the incidence of AEs (coded using the Medical Dictionary for Regulatory Activities dictionary), the incidence of severe asthma exacerbations, vital signs (blood pressure, pulse rate [measured at around 30 minutes post-dose, i.e. around the time of maximal plasma concentration (T_max_)], pre-dose ECG), haematology and clinical chemistry measures, measurement of serum cortisol, and routine liver function assessments.

### Statistical analysis

The study was powered for comparison of VI and SAL with placebo; the study was not designed to assess differences between VI and SAL. Sample size was calculated based on the primary endpoint and nominated powered secondary endpoint. The sample size of 330 (110 patients per arm) was planned on the basis of an estimated 10% withdrawal rate to give 96% power to detect a 175 ml difference between VI 25 mcg and placebo in weighted mean FEV_1_ at a two-sided significance level of 0.05 with anticipated standard deviation of 325 ml.

The following were all analysed using an analysis of covariates model with effects due to baseline, region, sex, age and treatment group: 0–24 hours weighted mean serial FEV_1_ after 12 weeks, change from baseline in percentage of rescue-free and symptom-free 24-hour periods over the first 84 on-treatment days, individual serial FEV_1_ assessment data at Week 12, and change from baseline in AM and PM PEF for the 12-week treatment period. For the primary and powered secondary endpoints, treatment interaction-by-region was analysed. Time to ≥12% and ≥200 ml increase above baseline FEV_1_ was analysed using a Cox proportional hazards model with treatment group as the explanatory variable and baseline FEV_1_, region, sex and age as covariates, with additional sensitivity analysis by log-rank test. Responses to global assessment of change questionnaire after 4 and 12 weeks of treatment were assessed using logistic (proportional odds) regression with covariates of region, sex, age and treatment group to produce odds ratios for estimated treatment differences.

In order to account for multiplicity across the key endpoints, a step-down testing hierarchy was applied. This stipulated that statistical significance (*P* < 0.05) of the primary endpoint treatment comparison of once-daily VI 25 mcg to placebo was required in order for statistical significance of powered secondary endpoints to be inferred. If a statistically significant treatment difference in both the primary and powered secondary endpoints was found, testing would be performed on all remaining efficacy endpoints without further multiplicity adjustment.

## Abbreviations

AE: Adverse event; ANCOVA: Analysis of covariates; BD: Twice daily; CI: Confidence interval; ECG: Electrocardiogram; FEV_1_: Forced expiratory volume in 1 second; FF: Fluticasone furoate; ICS: Inhaled corticosteroid; ITT: Intent-to-treat; LABA: Long-acting beta_2_ agonist; LS: Least squares; OD: Once daily; PEF: Peak expiratory flow; PP: Per protocol; RAMOS: Registration and Medication Ordering System; SAL: Salmeterol; SD: Standard deviation; SE: Standard error; VI: Vilanterol.

## Competing interests

JL has served as a consultant to and received lecture fees from AstraZeneca, GlaxoSmithKline, Merck Sharpe and Dohme, Novartis and UCB Pharma; has been partly covered by some of these companies to attend previous scientific meetings including the ERS and the AAAAI; has provided expert testimony for Barr Pharmaceuticals; and has participated in clinical research studies sponsored by AstraZeneca, GlaxoSmithKline, Merck Sharpe and Dohme, and Novartis. EDB has served as a consultant to AlkAbello, Almirall, Cephalon, Hoffman la Roche, ICON and MS Consulting Group; been on advisory boards for Almirall, AstraZeneca, Boehringer Ingelheim, Elevation Pharma, Forest, GlaxoSmithKline, Merck, Napp, Novartis and Nycomed; and received lecture fees from AlkAbello, AstraZeneca, Boehringer Ingelheim, Chiesi, GlaxoSmithKline, Novartis, Pfizer and Takeda; and his institution has received remuneration for participation in clinical trials sponsored by Actelion, Aeras, Almirall, AstraZeneca, Boehringer Ingelheim, Forest, GlaxoSmithKline, Hoffman La Roche, Merck, Novartis, Takeda and TEVA. WWB has served as a consultant to Amgen, AstraZeneca, Boehringer Ingelheim, Genentech, GlaxoSmithKline, MedImmune, Novartis and TEVA; served on advisory boards for Altair, Amgen, Centocor, GlaxoSmithKline, Johnson & Johnson, Merck Sharpe and Dohme, and Pfizer; received lecture fees from Merck Sharpe and Dohme; and received research funding from AstraZeneca, Ception, GlaxoSmithKline, MedImmune and Novartis. PMO’B has served as a consultant to AstraZeneca, Almirall, Boehringer Ingelheim, GlaxoSmithKline and Merck; has served on advisory boards for AIM, Altair, Boehringer Ingelheim, GlaxoSmithKline, MedImmune and Merck; has received lecture fees from Chiesi; and has received research funding from Amgen, AstraZeneca, Asmacure, Genentech and Ono. AW has served as a consultant to Almirall, Chiesi, Cytos and GlaxoSmithKline; and has received lecture fees and research grants from GlaxoSmithKline. ERB has served as a consultant to AstraZeneca, Boehringer Ingelheim, Genentech, GlaxoSmithKline, Johnson and Johnson, and Merck; and has performed clinical trials for AstraZeneca, Boehringer Ingelheim, Cephalon, Forest, Genentech, GlaxoSmithKline, KalaBios, MedImmune, Novartis, and Sanofi-Aventis which have been administered by his employer Wake Forest University School of Medicine. LJ, WTT and CG are employees of and hold stock in GlaxoSmithKline.

## Authors’ contributions

All authors, including the independent steering committee (JL, EDB, WWB, PMO’B, AW, ERB) together with authors employed by the sponsor (WTT, LJ, CG) had full access to the data and were responsible for the decision to publish the paper. All authors have seen and approved the final version of this manuscript for submission.

## Supplementary Material

Additional file 1 Patient demographics and baseline characteristics by country, ITT population. Click here for file

Additional file 2 Change from baseline in daily PM and AM PEF at Week 12, ITT population. Click here for file

Additional file 3 Statistical analysis of responders based on Global Assessment of Change Questionnaire, ITT population. Click here for file

Additional file 4 List of investigators and IECs/IRBs for B2C112060. Click here for file
