# Supplementary material for: Comparison of vilanterol, a novel long-acting beta2 agonist, with placebo and a salmeterol reference arm in asthma uncontrolled by inhaled corticosteroids
Source: J Negat Results Biomed. 2014 Jun 13;13:9. doi: 10.1186/1477-5751-13-9 (PMC4055937; doi:10.1186/1477-5751-13-9)
Supplement: Additional file 4 — List of investigators and IECs/IRBs for B2C112060. [file 1477-5751-13-9-S4.pdf]

CONFIDENTIAL

LIST OF INVESTIGATORS AND IECS/IRBS FOR B2C112060

Investigator	Sub-Investigator	Investigator no./Centre no.	Description of Research Facility, Hospital/ Institution, and Address	Name of IEC/IRB Committee, Address, Committee Chair
**Germany**				
Kappeler, Dominik. MD.	Meyer, Thomas. MD.	157619/ 081996	Inamed Research GmbH & Co. KG<br>Robert-Koch-Allee 29<br>82131 Gauting<br>Germany	Ethik-Kommission der Aerztekammer Schleswig-Holstein,<br>Bismarckallee 8 – 12, Bad Segeberg, 23795, Germany<br><br>Chairman Person: Ziegler, Albrecht
Kornmann, Oliver. MD.	Eich, Andreas. MD.<br>Michels, Berthold. MD.	068693/081997	IKF Pneumologie GmbH & Co. KG,<br>Stresemannallee3, Frankfurt, 60596, Germany	Ethik-Kommission der Aerztekammer Schleswig-Holstein,<br>Bismarckallee 8 – 12, Bad Segeberg, 23795, Germany<br><br>Chairman Person: Ziegler, Albrecht
Korn, Stephanie. MD.	Huebner, Marisa<br><br>Ruff, Andrea. MD.	122918/081998	Johannes-Gutenberg-Universitaet Mainz,<br>Geb. 406; 2. OG re.,<br>Langenbeckstr. 1, Mainz, 55131,	Ethik-Kommission der Aerztekammer Schleswig-Holstein,<br>Bismarckallee 8 – 12, Bad

Investigator	Sub-Investigator	Investigator no./Centre no.	Description of Research Facility, Hospital/ Institution, and Address	Name of IEC/IRB Committee, Address, Committee Chair
			Germany	Segeberg, 23795, Germany<br><br>Chairman Person: Ziegler, Albrecht
Watz, Henrik. MD.	Kirsten, Anne-Marie. MD.<br>Kretschmar, Gunther. MD.	101399/081999	Krankenhaus Grosshansdorf, Woehrendamm 80, Grosshansdorf, 22927, Germany	Ethik-Kommission der Aerztekammer Schleswig-Holstein,<br>Bismarckallee 8 – 12, Bad Segeberg, 23795, Germany<br><br>Chairman Person: Ziegler, Albrecht
**Peru**				
Cámere, Marco. MD.	Ajito, Daniel. MD.<br>Cámere, Diego. MD.<br>Mendoza, Ramón. MD.	075687/083079	Clínica San Gabriel,<br>Av. La Marina 2955,<br>San Miguel, Lima 32,<br>Peru	Comite Institucional de Bioetica (CIS) de Via Libre,<br>Jr. Paraguay 478, Lima Cercado, , Peru<br><br>Chairman Person: Mejia, Ada

Investigator	Sub-Investigator	Investigator no./Centre no.	Description of Research Facility, Hospital/ Institution, and Address	Name of IEC/IRB Committee, Address, Committee Chair
Guerra, Frances. MD.	Mori, Jose. MD.	021793/082738	Clínica Anglo Americana,<br>Av. Alfredo Salazar 350, Lima, Lima 27, Peru	Comite Institucional de Bioetica (CIS) de Via Libre,<br>Jr. Paraguay 478, Lima Cercado, , Peru<br><br>Chairman Person: Mejia, Ada
Iberico, Carlos. MD.	Gonzalez, Juan. MD.<br>Hinojosa, Fredy. MD.	187846/084388	Hospital Alberto Sabogal Sologuren – ESSALUD,<br>Jiron Colina 1081,<br>Callao, Callao 2,peru	Comité de Bioética del Hospital Alberto Sabogal,<br>Jr. Colina 1081, Callao 02, , Peru<br><br>Chairman Person: Gutiérrez Navarro, María del Rosario
Piñeiro, Andres. MD	Flores, Christina MD	021790/083032	Clínica El Golf,<br>Av. Aurelio Miro Quesada N° 1048, San Isidro, Lima 27, Peru	Comite Institucional de Bioetica (CIS) de Via Libre,<br>Jr. Paraguay 478, Lima Cercado, , Peru<br><br>Chairman Person: Mejia, Ada
Villarán, Cesar. MD.	Maekawa, Rosalba<br>Morello, Enrique<br>Tsukayama, Miguel. MD<br>Vasquez, Natalia. MD.	021792/082739	Clínica Ricardo Palma,<br>Av. Javier Prado Este 1010, Lima 27, Peru	Comite Institucional de Bioetica (CIS) de Via Libre,<br>Jr. Paraguay 478, Lima Cercado, , Peru

Investigator	Sub-Investigator	Investigator no./Centre no.	Description of Research Facility, Hospital/ Institution, and Address	Name of IEC/IRB Committee, Address, Committee Chair
				Chairman Person: Mejia, Ada
**Poland**				
Hajol, Elzbieta. MD.	None	215936/083447	NZOZ KRAK-MEDYK Sp. z o. o.,<br>Ul. Ulanow 29, Krakow, 31-455,<br>Poland.	Komisja Bioetyczna przy Okregowej Izbie Lekarskiej,<br>Ul. Krupnicza 11a, Krakow, 31-123, Poland<br><br>Chairman Person: Janikowski, Mariusz
Harat, Rafal. MD.*	Krok, Bogdan. MD.<br>Lorecik, Magdalena. MD<br>Plachcinski, Michal. MD.<br>Zygmunt, Katarzyna. MD.	215937/083534	Medex,<br>Ul. Kubusia Puchatka 2,<br>Chrzanow, 32-500,<br>Poland.	Komisja Bioetyczna przy Okregowej Izbie Lekarskiej,<br>Ul. Krupnicza 11a, Krakow, 31-123, Poland<br><br>Chairman Person: Janikowski, Mariusz
Niemiec, Artur. MD.	Kotucha, Wojciech. MD.<br>Pajor, Adam. MD.	215938/083449	5 Wojskowy Szpital Kliniczny,<br>Ul. Wroclawska 1-3,<br>Krakow, 30-901, Poland.	Komisja Bioetyczna przy Okregowej Izbie Lekarskiej,<br>Ul. Krupnicza 11a, Krakow, 31-123, Poland<br><br>Chairman Person: Janikowski, Mariusz

Investigator	Sub-Investigator	Investigator no./Centre no.	Description of Research Facility, Hospital/ Institution, and Address	Name of IEC/IRB Committee, Address, Committee Chair
Pulka, Grazyna. MD.	Soja, Jerzy. MD.	001325/083176	ALL-MED,<br>Ul. Sw. Marka 31/IU, Krakow, 31-023, Poland.	Komisja Bioetyczna przy Okregowej Izbie Lekarskiej,<br>Ul. Krupnicza 11a, Krakow, 31-123, Poland<br><br>Chairman Person: Janikowski, Mariusz
Wolak-Sobiczewska, Lucyna. MD/PhD.	Horaczynska-Wojtas, Anna. MD.<br>Krupa-Borek, Izabella. MD./PhD	194725/086606	Centrum Alergologii NZOZ,<br>Ul. Kawaleryjska 10, Lublin,<br>20-552, Poland.	Komisja Bioetyczna przy Okregowej Izbie Lekarskiej,<br>Ul. Krupnicza 11a, Krakow, 31-123, Poland<br><br>Chairman Person: Janikowski, Mariusz
**Ukraine**				
Blazhko, Viktor. MD.	Dementieva, Iana. MD.<br>Tymchenko, Ganna. MD.<br>Zamaziy,<br>Antonina. MD./PhD	189617/084840	City Clinical Hospital #13,<br>Department of Pulmonology # 2,<br>137, Gagarina Str. Kharkiv, 61124, Ukraine.	Central Ethics Committee of Ministry of Health of Ukraine,<br>5-Narodnogo opolchennia Str, Kyiv, 03680, Ukraine<br><br>Chairman Person: Kornatskyi, Vasyl

Investigator	Sub-Investigator	Investigator no./Centre no.	Description of Research Facility, Hospital/ Institution, and Address	Name of IEC/IRB Committee, Address, Committee Chair
Feshchenko, Yuriy. MD./PhD	Adamchuk, Oleksandr. MD<br>Kanarskyi, Alexandr. MD<br>Korchynska, Maryna. MD.<br>Kuryk, Lesya. MD./PhD	001069/084835	Institute of Phthisiatry and Pulmonology,<br>Department of Pulmonology, 10, Amosova Str, Kiev,03680, Ukraine	Central Ethics Committee of Ministry of Health of Ukraine, 5-Narodnogo opolchennia Str, Kyiv, 03680, Ukraine<br><br>Chairman Person: Kornatskyi, Vasyl
Iashyna, Liudmyla. MD.	Ishchuk, Svetlana. MD.<br>Polianska, Marina. MD./PhD<br>Savelieva, Liudmila. MD.	001071/084836	Institute of Phthisiatry and Pulmonology,<br>Lung diseases diagnostics, therapy and clin.pharm., 10, M.Amosova str., Kyiv, 03680, Ukraine	Central Ethics Committee of Ministry of Health of Ukraine, 5-Narodnogo opolchennia Str, Kyiv,0 3680, Ukraine<br><br>Chairman Person: Kornatskyi, Vasyl
Korzh, Oleksii. MD./PhD	Krasnokutskiy, Sergiy. MD.<br>Lazarenko, Anastasiia. MD.<br>Liashok, Artem. MD.<br>Lobortas, Oksana. MD.<br>Vaskiv, Nataliia. MD	164525/084839	Kharhiv Multidisciplinary Clinical Hospital №17,<br>Department of therapy,<br>195 Moskovskii Avenue,<br>Kharkiv, 61037, Ukraine	Central Ethics Committee of Ministry of Health of Ukraine, 5-Narodnogo opolchennia Str, Kyiv, 03680, Ukraine<br><br>Chairman Person: Kornatskyi, Vasyl

Investigator	Sub-Investigator	Investigator no./Centre no.	Description of Research Facility, Hospital/ Institution, and Address	Name of IEC/IRB Committee, Address, Committee Chair
Monogarova, Nadiya. MD./PhD.	Bilokon, Tamila. MD.<br>Noreyko, Victoriya. MD./PhD.<br>Semendyayeva, Olena. MD.	001063/084832	Donetsk Regional Territory Medical Association,<br>Department of Pulmonology, 14 Illicha avenue, Donetsk, 83099, Ukraine	Central Ethics Committee of Ministry of Health of Ukraine, 5-Narodnogo opolchennia Str, Kyiv, 03680, Ukraine<br><br>Chairman Person: Kornatskyi, Vasyl
Ostrovskyy, Mykola. MD./PhD.	Kulynych, Marianna. MD.<br>Savelikhina, Iryna<br>Varunkiv, Oleksandr. MD.	214594/084841	Regional Centre of Phthisiology and Pulmonology,<br>Department of Pulmonology,<br>17 I.Franka, Str. Ivano-Frankivsk, 76018, Ukraine	Central Ethics Committee of Ministry of Health of Ukraine, 5-Narodnogo opolchennia Str, Kyiv, 03680, Ukraine<br><br>Chairman Person: Kornatskyi, Vasyl
Panina, Svitlana. MD./PhD.	Gondulenko, Nataliya. MD./PhD.<br>Sanina, Nataliya. MD.	135595/084834	Ukrainian State Institute of Medical and Social Problems of Disability, research therapeutic department, 1a Radyansky Str., Dnipropetrovsk, 49027, Ukraine	Central Ethics Committee of Ministry of Health of Ukraine, 5-Narodnogo opolchennia Str, Kyiv, 03680, Ukraine<br><br>Chairman Person: Kornatskyi, Vasyl
Pertseva, Tetyana. MD./Phd	Bogats'ka, Kateryna. MD./PhD<br>Gashynova, Kateryna.	001059/084833	City Clinical Hospital #6,<br>Department of therapy with pulmonological wards, Batumska str	Central Ethics Committee of Ministry of Health of Ukraine, 5-Narodnogo opolchennia Str,

Investigator	Sub-Investigator	Investigator no./Centre no.	Description of Research Facility, Hospital/ Institution, and Address	Name of IEC/IRB Committee, Address, Committee Chair
	MD./PhD		13., Dnipropetrovsk, 49074, Ukraine	Kyiv, 03680, Ukraine<br><br>Chairman Person: Kornatskyi, Vasyl
Soldatchenko, Sergiy. MD./PhD.	Dudchenko, Leyla. MD.<br>Korzhevnyk, Iryna. MD.<br>Maslikova, Galina. MD.	200280/084842	Research Institute of Physical Methods of Treatment and Medical Climatology, Department of Pulmonology, 8, Mukhina Str., Yalta, 98603, Ukraine	Central Ethics Committee of Ministry of Health of Ukraine, 5-Narodnogo opolchennia Str, Kyiv, 03680, Ukraine<br><br>Chairman Person: Kornatskyi, Vasyl
Zorin, Valeriy. MD./PhD.	Plastun, Mariia. MD.<br>Shabalin, Yuriy. MD	191363/084838	Simferopol Railroad Hospital, Therapy and Allergology Department, 142, Kyivska str. 95043, Simferopol, Ukraine	Central Ethics Committee of Ministry of Health of Ukraine, 5-Narodnogo opolchennia Str, Kyiv, 03680, Ukraine<br><br>Chairman Person: Kornatskyi, Vasyl
**United States**				
Bernstein, David. MD.	Bernstein, Jonathan. MD.<br>Evans, Sherry. CNP	018980/080002	Bernstein Clinical Research Center, 8444 Winton Road, Cincinnati, 45231, Ohio, United States	Quorum Review, Inc.,<br>1601 Fifth Avenue,<br>Seattle, 98101, United States<br><br>Chairperson: David, Kelley

Investigator	Sub-Investigator	Investigator no./Centre no.	Description of Research Facility, Hospital/ Institution, and Address	Name of IEC/IRB Committee, Address, Committee Chair
Boscia, III, Joseph. MD.	Benfield, Meredith. RRT, CCRC.<br>Buice, Jane N. RN, CCRC.<br>DeLaCruz, Luis. MD.<br>Dove, Tracy. CRC<br>Duncan, Bonnie. RTR, CRC.<br>Feldman, Gregory. MD.<br>Hughes, Richard. MD.<br>Raynor, Anthony. PA-C.<br>Shetley, Carrie. CST, CRC.<br>Sprinkle, Brenda. PA-C.<br>Stockinger, Helen. MD.<br>Williams, Haley. RRT, CCRC.<br>Abercrombie, Wendy. FNP-BC	067189/080003	CU Pharmaceutical Research,<br>1Medical Park Drive,<br>Chester, 29706, South Carolina, United States	Quorum Review, Inc.,<br>1601 Fifth Avenue,<br>Seattle, 98101, United States<br><br>Chairperson: David, Kelley
Cole, Jeremy. MD.	Thurman, Louise. MD. MPH.	040688/080004	IPS Research Company,<br>1111 North Lee, Suite 400,<br>Oklahoma City, 73103, Oklahoma, United States	Quorum Review, Inc.,<br>1601 Fifth Avenue,<br>Seattle, 98101, United States<br><br>Chairperson: David, Kelley

Investigator	Sub-Investigator	Investigator no./Centre no.	Description of Research Facility, Hospital/ Institution, and Address	Name of IEC/IRB Committee, Address, Committee Chair
Cruz, Humberto. MD.	Fleites, Norberto, MD.	175852/079836	Florida Institute for Clinical Research,<br>7200 Curry Ford Road, Orlando, 32822, Florida, United States	Quorum Review, Inc.,<br>1601 Fifth Avenue,<br>Seattle, 98101, United States<br><br>Chairperson: David, Kelley
Dunn, Karen. MD.	Aarons, Alan. MD.<br>Doherty, Margaret. PA-C.<br>Firrincieli, Vincent. MD.<br>LaForce, Craig. MD.	019942/080005	North Carolina Clinical Research, Twin Lakes Professional Center, 2615 Lake Drive, Suite 301, Raleigh, 27607, North Carolina, United States	Quorum Review, Inc.,<br>1601 Fifth Avenue,<br>Seattle, 98101, United States<br><br>Chairperson: David, Kelley
Feldman, Gregory. MD, CPI.*	Boscia, III, Joseph. MD, FCCP, CPI.<br>Buice, Anna<br>Jolly, Laura<br>Raynor, Anthony, PA-C.<br>Rumfelt, Gail. CMA, CPC.<br>Rumfelt, Lauren<br>Weirick, Brian, DO.<br>Abercrombie, Wendy. FNP-BC	061057/082576	S. Carolina Pharmaceutical Research, 500 Plaza Circle Dr., Clinton, 29325, South Carolina, United States	Quorum Review, Inc.,<br>1601 Fifth Avenue,<br>Seattle, 98101, United States<br><br>Chairperson: David, Kelley
Pedinoff, Andrew. MD.	Caucino, Julie. DO.<br>Sikorski, Kristen. MD.<br>Skolnick, Helen. MD.	017957/081358	Princeton Center for Clinical Research,<br>Montgomery Professional Parks,<br>24 Vreeland Drive, Skillman, 8558,	Quorum Review, Inc.,<br>1601 Fifth Avenue,<br>Seattle, 98101, United States

Investigator	Sub-Investigator	Investigator no./Centre no.	Description of Research Facility, Hospital/ Institution, and Address	Name of IEC/IRB Committee, Address, Committee Chair
	Southern, Darrell. MD.		United States	Chairperson: David, Kelley
Qaqundah, Paul. MD.	Christensen, Jennifer. MD.<br>Cornelsen, Ronald. MD.<br>Sarrouf, John. DO.<br>Tolly, Kristy. MD.	007905/079834	Pediatric Care Medical Group, Inc, 17822 Beach Boulevard, Huntington Beach, 92647, California, United States	Quorum Review, Inc.,<br>1601 Fifth Avenue,<br>Seattle, 98101, United States<br><br>Chairperson: David, Kelley
Saff, Ronald. MD.	Stabley, Christine. PA-C	014995/079833	Allergy & Asthma Diagnostic Treatment Center,<br>2300 Centerville Road, Tallahassee, 32308, United States	Quorum Review, Inc.,<br>1601 Fifth Avenue,<br>Seattle, 98101, United States<br><br>Chairperson: David, Kelley
Sterling, Richard. MD.	None	017515/079835	Sterling Ear, Nose & Throat, PA,<br>2221 St. Matthews Road,<br>Orangeburg, 29118	Quorum Review, Inc.,<br>1601 Fifth Avenue,<br>Seattle, 98101, United States<br><br>Chairperson: David, Kelley
Weinberg, Paul. MD.	Brown, Chevy. MPH, CCRC.<br>Feldman, David. BBA, CCRC.<br>Fisher, Amy<br>Garimella, Prasad. MD.<br>Gross, Suvi. BA.	017249/082866	Gwinnett Biomedical Research,<br>Suite 160,<br>600 Professional Drive,<br>Lawrenceville, 30046, United States	Quorum Review, Inc.,<br>1601 Fifth Avenue,<br>Seattle, 98101, United States<br><br>Chairperson: David, Kelley

Investigator	Sub-Investigator	Investigator no./Centre no.	Description of Research Facility, Hospital/ Institution, and Address	Name of IEC/IRB Committee, Address, Committee Chair
	Hain, Aldin. BSBA, CNA.<br>Kaplan, Larry (Lawrence). MD.<br>Lozynsky, Regina. LPN.<br>McGann, Jr., William. MD.<br>Nisbet, Rachel. MD.<br>Sineway, Michael. MD.<br>Weinberg, Amanda. BA<br>Jasani, Rajesh. MD.<br>Brown, George. Jr., BS.			
Weinstein, Steven. MD.	Van Lare, Casey. CFNP.	011820/081554	Allergy & Asthma Specialists Medical Group, 17742 Beach Boulevard, Huntington Beach, 92647, California, United States.	Quorum Review, Inc.,<br>1601 Fifth Avenue,<br>Seattle, 98101, United States<br><br>Chairperson: David, Kelley

All centres participated in the study under the US IND.
*No Patients Enrolled
